# Mindfulness practices for alleviating distress among postpartum women

**DOI:** 10.1590/0034-7167-2024-0525

**Published:** 2025-12-08

**Authors:** Fernanda Veras Vieira Feitosa, Raimunda Magalhães da Silva, Waleska Benicio de Oliveira Carvalho, Débora Pereira Paixão, Marta Maria Soares Herculano, Maria Helena Carvalho Valente Presado, Vânia Krisna de Oliveira Albuquerque, Laura Pinto Torres de Melo

**Affiliations:** I Universidade de Fortaleza. Fortaleza, Ceará, Brazil; II Universidade Federal do Ceará. Fortaleza, Ceará, Brazil; III Escola Superior de Enfermagem de Lisboa. Lisboa, Portugal; IV Universidade Federal do Ceará, Empresa Brazileira de Serviços Hospitalares. Fortaleza, Ceará, Brazil

**Keywords:** Mindfulness, Postpartum Period, Women, Resilience, Psychological, Mental Health., Atención Plena, Periodo Posparto, Mujeres, Resiliencia Psicológica, Salud Mental.

## Abstract

**Objectives:**

to understand how mindfulness practice helps alleviate distress among postpartum inpatients at a public maternity hospital.

**Methods:**

this intervention study was carried out between March and June 2022 with 19 postpartum women. Data were collected through interviews conducted before and after eight mindfulness sessions and analyzed using thematic analysis.

**Results:**

mindfulness fostered self-regulation, attentional control, emotion regulation, a change in perspective on the self, and increased body awareness. It also reduced fear, anxiety, and stress.

**Final Considerations:**

by becoming aware of the physical and emotional sensations of their bodies, postpartum women engaged in a reflective process that helped alleviate distress during the postpartum period.

## RESUMEN

**Objetivos:**

comprender las implicaciones de la práctica de mindfulness para superar las inquietudes de mujeres en el puerperio hospitalizadas en una maternidad pública

**Métodos:**

estudio de intervención realizado en una maternidad entre marzo y junio de 2022, con 19 mujeres puérperas. Los datos se recogieron mediante entrevistas aplicadas antes y después de ocho intervenciones de mindfulness. Los resultados se organizaron y analizaron a través del análisis temático.

**Resultados:**

la práctica fomentó en las puérperas la autorregulación psicológica, el control de la atención, la regulación emocional, cambios en la autopercepción y una mayor conciencia corporal. Asimismo, redujo temores, angustias y estrés.

**Consideraciones Finales:**

al despertar la conciencia hacia las sensaciones físicas y emocionales de sus cuerpos, las mujeres en el puerperio se involucraron en un proceso reflexivo que facilitó la superación de las inquietudes propias de esta etapa.

## INTRODUCTION

A maternal death occurs worldwide every minute, totaling about 600,000 deaths annually. In highand middle-income countries, the maternal mortality rate exceeds 12 per 100,000 live births, while in low-income countries it reaches 239 per 100,000 live births. To address this issue, the United Nations (UN) and the World Health Organization (WHO) set global targets in the Sustainable Development Goals (SDGs) for 2030 to reduce maternal and neonatal mortality and adverse outcomes across the pregnancy-postpartum cycle^(1)^.

Castiglioni^(2)^ defines the postpartum period as a stage in the pregnancy-postpartum cycle that represents the transition to motherhood and the return of the body to its pre-pregnancy state. This period is characterized by numerous physical transformations and emotional adjustments, which may result in adversities affecting the mother-child bond.

During this period, women face conflicts between pregnancy expectations and postpartum changes, leading to emotional vulnerabilities. The postpartum period is also characterized by non-obstetric conditions, including mental disorders and noncommunicable diseases, which significantly affect the lives of postpartum women^(3,4)^.

Thus, when complications occur before or after birth, motherhood may be disrupted or altered by physical and psychological stress. This can lead to changes in the care provided to their infants and in how women perceive themselves and relate to their surroundings, requiring internal and external adjustments to better cope with the situation^(5)^.

In this context, progress has been made in developing therapeutic alternatives to mitigate the effects of the postpartum period and its complications. Among these alternatives are complementary and alternative medicine (CAM) practices, which provide comprehensive care and include herbal medicine, acupuncture, homeopathy, mindfulness, and balneotherapy, among others. Introduced in 2006 under the National Plan for Integrative and Complementary Practices, these services expanded to 29 modalities by 2018^(6)^.

Mindfulness can be an appropriate and effective approach: it involves deliberate, nonjudgmental awareness that reduces the emergence of harmful thoughts and feelings that adversely affect health. The practice aims to foster self-regulation through mindful attention, a form of meditation that supports emotional balance and bodily self-awareness. It can improve sleep quality and promote psychological, physical, and interpersonal well-being, among other benefits^(7-9)^.

In high-income countries, mindfulness has been applied across diverse clinical settings, benefiting both body and mind. It is widely used to promote behavior change and has demonstrated positive effects in adults with a range of health conditions^(10,11)^.

Given the multiple nuances of the postpartum period and the implications for women’s health following complications in this phase, mindfulness may be a valuable tool. Accordingly, we sought to explore the changes that mindfulness practice produces in postpartum inpatients at a public maternity hospital.

Although mindfulness is increasingly recognized for its benefits in mental health, qualitative studies examining its application during the postpartum period in high-risk hospital settings in Brazil remain scarce. Little is known about the experiences of hospitalized postpartum women after mindfulness-based interventions, particularly regarding emotional, bodily, and interpersonal aspects during this period. This gap hinders the development of woman-centered care practices integrated with the specific demands of perinatal care in adverse situations.

We expect the study results to prompt new reflections on postpartum care and to provide evidence to rethink and reassess policies aimed at comprehensive care and women’s well-being, thereby promoting environments of safety, respect, and holistic support.

## OBJECTIVES

To understand the implications of mindfulness practice for alleviating the distress experienced by postpartum inpatients at a public maternity hospital.

## METHODS

### Ethical aspects

The institution’s Research Ethics Committee approved the study. Ethical guidelines for research involving human participants were followed in accordance with Resolutions 466/2012 and 510/2016 of the National Health Council (CNS)^(12,13)^.

After institutional approval, participants were approached in person, informed about the study’s purpose and procedures, and signed the Informed Consent Form (ICF). Because admissions were continuous, information and consent procedures were provided on an ongoing basis. Importantly, no invited participant declined to participate, and none had any ties to the researchers.

To protect confidentiality, participants were assigned codes (the initials “PW” for “postpartum women” followed by a number indicating interview order). Interviews were concluded based on data saturation and when participants’ accounts had been adequately captured and understood.

### Theoretical-methodological framework

The study was grounded in the theoretical and methodological tenets of phenomenology, with emphasis on the concepts of Edmund Husserl^(14)^ and Maurice Merleau-Ponty^(15)^.

According to Husserl, experience is a mode of relation by which a subject engages with objects in their environment. This relation should not be reduced to mere manifestations of internal mental states, since it is always intentional and directed toward something external - the “phenomenon” - that is, manifestations of consciousness in relation to the context in which it is immersed^(16)^.

Merleau-Ponty^(15)^ locates experience in perception. For him, perception restores the logic of life and opens access to the world in which one participates. It is through processes of understanding that the primordial meanings of the latent truth of lived experience emerge. Perceiving, therefore, is an action - a pragmatic form of knowing in relation to the phenomenon.

Merleau-Ponty^(15)^ further argues that people are immersed in the world through the body: it is through the body that they perceive and inhabit the world. The body is the ground for meaning-making - the basis on which relations between self and world acquire significance. Nevertheless, the self is apprehended in its totality only as it is structured through its relation with the environment, which in turn is reflected back on that environment. Viewing people as embodied beings underscores the importance of perceptual experience and supports the view that knowledge begins in the body and in relation to the lived world.

### Study design

We conducted an intervention study using a qualitative approach. The study adhered to the Consolidated Criteria for Reporting Qualitative Research (COREQ). Non-quantifiable data were collected based on participants’ lived experiences and were captured through the subjective characteristics of each person involved in the studied setting^(17,18)^.

### Methodological procedures

For the mindfulness intervention, participants were grouped into cohorts of four to ten postpartum women, a size recommended by Zhang^(5)^ for conducting group processes. This configuration encouraged opportunities to speak and facilitated a better understanding and capture of the phenomena expressed. Each participant attended eight mindfulness sessions.

Among mindfulness-based interventions, Mindfulness-Based Cognitive Therapy (MBCT) and Acceptance-Based Behavioral Therapy (ABBT) are commonly used. This study adopted the Mindfulness-Based Stress Reduction (MBSR) program, developed by Kabat-Zinn and structured in eight sessions^(19-21)^.

Mindfulness sessions were offered three times weekly during morning and afternoon sessions, according to a prearranged schedule. The sequence of sessions is described below.

In the first session, participants practiced Mindful Breathing (focused attention on the breath). This technique uses the breath as an anchor, directing attention to the sensations of “breathing with awareness” - that is, moment-to-moment awareness^(22)^.

The second session involved a Body-scan practice, which aimed to anchor attention on any sensation arising in the resting body. Sensations - pleasant, unpleasant, or neutral - were acknowledged and allowed to arise and pass, cultivating openness, curiosity, and kindness, which are fundamental attitudes in mindfulness^(19)^.

In the third session, participants practiced Walking Meditation to bring the here-and-now into movement: the exercise cultivates attention on the moving body and on what it is like to walk without an intention to reach a destination^(20)^.

The fourth session consisted of an Open-monitoring practice (reflection on family and social relationships). This practice involves witnessing the arising, unfolding, and cessation of mental events in our field of experience and developing the ability to observe mental events without attachment or aversion^(23)^.

In the fifth session, participants practiced Mindful Movement, focusing on bodily emotional responses, impulses to avoid or escape discomfort, self-judgment, and evaluative tendencies^(21)^.

In the sixth session, participants engaged in an Interdependence practice, reflecting on relationships across past, present, and future. The exercise aimed to broaden motivation to expand the circle of care, foster connections, reduce indifference toward others, and cultivate gratitude and contentment with life^(22)^.

In the seventh session, participants practiced Loving-kindness (metta), with exercises focused on relations with the self and with others. The session sought to create a space that connects people with their hearts and raises awareness of caring for one another^(20)^.

The eighth session consisted of a Recognition of Shared Humanity practice, accompanied by expressions of goodwill. This session aimed to increase empathy, connection, and a sense of belonging, and to cultivate attitudes of goodwill^(20,22)^.

### Study setting

This study was conducted in a Pregnancy-Baby-Postpartum Care Home (*Casa da Gestante, Bebê e Puérpera* - CGBP) affiliated with a tertiary-level public maternity hospital specialized in high-risk obstetrics. The CGBP functions as a residential facility providing daily, specialized care and follow-up for high-risk pregnant women, postpartum women, and newborns who require ongoing health services but do not require hospital admission.

The facility operates as an annex of the maternity hospital and is staffed by a multidisciplinary team providing specialized care. The team includes physicians, nurses, nursing technicians, psychologists, physiotherapists, and other professionals who are available on call.

### Data source

We recruited 19 postpartum women using convenience sampling. Inclusion criteria were postpartum women aged 18 years or older who were able to remain in the unit for ten days^(10)^ to participate in the eight mindfulness sessions and in the preand post-intervention interviews. Women requiring absolute bed rest by medical order or those unable to complete all study procedures were excluded.

Before each interview, we reserved time for informal conversation to help overcome potential barriers to participation; however, these exchanges were brief given the facility’s service schedule.

### Data collection and organization

Data were collected between March and June 2022 through semi-structured interviews to obtain sociodemographic, clinical, obstetric, and lifestyle information. The psychologist-researcher and two trained field research assistants conducted the interviews.

The data collection phase comprised three stages: (1) an initial interview with guided questions about postpartum experiences and expectations regarding the mindfulness practice; (2) participation in eight mindfulness sessions in progressive stages^(8)^, each lasting approximately 60 minutes and delivered in groups of up to ten women in a prepared setting; and (3) a follow-up interview addressing experiences after the intervention and its implications. Both interviews (preand post-intervention) were completed by all 19 participants (no dropouts).

Interviews were audio-recorded individually: the initial interview took place before the intervention and the final interview after it, with durations ranging from 20 to 60 minutes according to each participant’s needs. To ensure reliability, we transcribed the interviews verbatim and returned them to participants for verification (member checking).

### Data analysis

We analyzed the data using thematic analysis to organize the participants’ accounts through stages of pre-analysis, exploration, and interpretation. We sought convergent and divergent perspectives that were meaningful to the postpartum women. Through iterative close readings of the material, we identified meaning units related to the lived phenomenon^(18)^. Interpretations were informed by phenomenological approaches.

The dataset was organized into three themes: “Expectations of change from the mindfulness practice”, “Implications of mindfulness practice for body and mind”, and “Mindfulness in alleviating postpartum distress experienced by women”.

## RESULTS

Of the 19 postpartum women enrolled in the study (n = 19), 7 (35.0%) lived in Fortaleza and 12 (65.0%) in the metropolitan area. Participants’ ages ranged from 18 to over 36 years; the most frequent age group was 21-25 years (n = 6; 31.6%). Regarding serological testing, 16 women (84.2%) had reactive VDRL results, and 13 newborns (68.4%) were diagnosed with congenital syphilis. Obstetric data showed that 10 participants (52.6%) were multiparous and 9 (47.4%) primiparous. Regarding mode of delivery, 15 women (78.9%) underwent cesarean section and 4 (21.1%) had vaginal deliveries.

Regarding feelings about hospitalization, 7 women (36.8%) reported feeling satisfied, 7 (36.8%) reported feeling fearful, and 3 (15.8%) reported feeling apprehensive (other responses or missing data accounted for the remainder). Concerning engagement in stress-reduction activities, 5 participants (25.0%) reported practicing such activities, while 14 (75.0%) did not.

### Expectations of change from the mindfulness practice

This theme addressed participants’ expectations regarding the mindfulness practice, including the desire to better cope with the psychological and bodily changes experienced during the postpartum period in a high-risk setting.

*I had planned the pregnancy, but it was a shock - my baby fell ill* [...] *I hope this practice will help calm my body and give me some relief. I keep thinking about my child all the time; it exhausts me. My mind needs calm.* (P1)

*I planned the pregnancy; I wasn’t prepared for a preterm birth. The postpartum period has been very hard. My mind is all over the place and my body doesn’t respond to what my head tells it. I hope this technique helps.* (P6)

*I’m anxious for all of this to pass. I hope this practice helps me calm down and find some peace.* (P10)

[...] *I need my body to be cared for. I’ve been struggling and I believe this will help me overcome many of those struggles. I long for a calm mind and a body free of tension.* (P17)

Participants hoped the mindfulness practice would create space for changes that extend beyond body and mind, improving family relationships, their relationship with themselves, and their experience during hospitalization, as reflected in these statements:

*I believe it will relax my mind and improve my relationship with my husband and with myself, because I need to work through things inside me.* (P8)

*Fear surrounds my mind and body because I feel guilty about infecting my baby. I want mindfulness to help me get past this so I can look at him without rejection.* (P5)

*I hope it brings benefits, relaxes my body, eases the pain, and helps calm us, because sometimes I feel desperate; I feel guilty and angry with everyone.* (P12)

*I need to pull myself together because I don’t feel like the same woman: my relationship with my family has changed. I think the hospitalization and this illness affected me; I need this activity to be effective.* (P16)

### Implications of mindfulness practice for body and mind

This theme addressed participants’ accounts after experiencing the mindfulness practice. The technique appeared to offer multiple benefits, creating a space of calm for the suffering and tensions that arise during the postpartum period.

For example, intense crying could be soothed - not because the reasons for distress vanished, but because new possibilities emerged that reduced anxiety and the burden of the situation. Participants were better able to disengage from intrusive thoughts and focus on a calmer state, as illustrated below:

*There are countless benefits: mental calm, relief from tension and stress during these challenging days. My body felt light; the crying calmed down and subsided. I stopped thinking the worst.* (P5)

*This practice truly benefits both mind and body. My tensions decreased and the swelling improved significantly. The breathing techniques lifted a weight off me. It’s amazing how much of a difference they made to my body. The crying that used to be uncontrollable now seems to have disappeared.* (P3)

*This practice calms me a great deal, shifting my focus away from negative things and lifting a weight off me. I feel it eases the burden on my conscience - I relax.* (P11)

Due to their circumstances, some postpartum women reported feeling aversion toward their babies, which caused suffering and guilt. However, after the interventions they reported improved relationships with their babies, feeling closer and changing how they related to them.

[...] *I couldn’t sleep at night; after those sessions I started sleeping. Now I’m calm, very relaxed. My relationship with my baby improved. He was calm when we practiced. Before, something felt wrong: I didn’t accept him, and I blamed myself for that.* (P13)

[...] *At first, I felt repulsion toward my baby, and it caused me pain. But after these activities, I noticed that things changed.* (P12)

[...] *Now I can sleep much better; I can relate to my daughter without guilt - it’s wonderful. This practice really changed many things in me.* (P15)

Notably, participants reported that mindfulness helped reduce physical pain. They mentioned swelling and bodily pain after delivery, which they attributed to tension and the typical changes of this phase. Mindfulness proved broadly beneficial both in reducing discomfort and in promoting a sense of calm in the face of the situation.

*These activities were a turning point, opening new ways of living and coping more gently, and reducing fears.* (P6)

[...] *My body relaxed: tensions and pains decreased, and the swelling improved. I believe it helped circulation. And my mind - how it improved! I had been very distressed.* (P8)

[...] *So many things changed in me, more than I could have imagined. My body and my thoughts were transformed by these processes.* (P12)

### Mindfulness in alleviating postpartum distress experienced by women

This theme addressed issues related to using mindfulness to cope with the fears and insecurities experienced during the postpartum period. Participants reported reductions in fear, anguish, and stress, and described assigning new meaning to their lived experiences.

*My mind calmed, and my body - so tense - found a place of calm amid so many dead ends and anxieties.* (P8)

*I was very agitated, adrift, and full of fear. Over time, my emotions found a place of peace inside me* […] *I calmed down.* (P12)

*When we meditate, we feel better and happier even while immersed in so much pain.* (P13)

Fear, pain, and despair gave way to hope and a deeper bond with the child, as the following statements illustrate:

*I realized these emotions only held me back. Mindfulness has been very helpful in facing the fears we were going through.* (P10)

*These days many things have taken on new meaning for us. I began to look at my baby without fear and pain, with hopeful eyes, believing he would be okay.* (P3)

*After these days I feel closer to my baby. I look at him without guilt and enjoy him more. I learned to calm myself and put fear and guilt aside - to see life with new eyes.* (P19)

## DISCUSSION

In the postpartum period, women face psychological distress stemming from bodily changes, caregiving demands, and personal and professional readjustments. In high-risk pregnancies, this burden is intensified, resulting in increased anxiety, stress, and fear^(24,25)^. These conditions are important contributors to maternal grief, as reflected in participants’ accounts.

Schiele^(25)^ and Araújo^(10)^ argue that concerns related to health problems, loss of control, isolation, separation from the family environment, physical discomfort, and obstetric procedures-many of which are physically and psychologically invasive-generate adverse emotional responses.

These emotions can be understood in light of Merleau-Ponty’s principles^(15)^: experience is inherent to the individual and cannot be completely suppressed. For Merleau-Ponty, experience exceeds what can be fully expressed in thoughts, gestures, or words; its ambiguous nature involves affective dimensions that resist full articulation and require an open interplay among body, mind, and subjectivity.

In this context, da Silva et al.^(26)^ and Porter et al.^(9)^ suggest that mindfulness may help address the challenges of the postpartum period by cultivating present-moment, nonjudgmental attention and by fostering bodily awareness of the lived moment. Klainin-Yobas et al.^(27)^ and Santos et al.^(28)^ contend that psychological resilience is a dynamic process: it involves change and enables individuals to find balance and to flourish in their relationships with themselves, others, and the world. Resilience is a product of human-environment interaction.

Participants in this study reported enhanced mental and physical resilience in the face of adversity. Merleau-Ponty^(15)^ argues that the body’s intentionality is directed toward the world as conceived by the subject, reflecting desires and meanings formed over the life course and embedded in the symbolic perspectives through which individuals live.

The use of mindfulness-based interventions (MBIs) in clinical settings has increased, and research highlights their positive effects on health. According to Silva et al.^(26)^ reports that such interventions are effective in reducing anxiety, stress, and depression during the perinatal period. The participants’ accounts support these findings: they reported marked reductions in perceived stress and increases in mindfulness.

Participants described experiencing mental calm, less anguish, and reduced tearfulness, underscoring the role of mindfulness in promoting well-being and mental health during the perinatal period. Wang at al.^(29)^ notes that the practice helps individuals become aware of their thoughts and feelings, enabling a more constructive relationship with them; this, in turn, facilitates self-acceptance and the capacity to cope with challenging health conditions such as hospitalization or serious illness.

The body builds its forms of knowledge and bodily meanings based on how it experiences the present moment. Thus, irrespective of the mode of expression, the body incorporates meaning and shapes the singularity of each person’s existence^(15,30)^.

Merleau-Ponty^(15)^ conceives behavioral changes in the body as a symbolic expression of existence. From his perspective, the body may withdraw from habitual transparency and become opaque, allowing sensations and emotions that are usually repressed to surface. When a mother imagines the situations her child faces during treatment, she experiences distinct bodily sensations; in that moment, the body is the site where her existence is manifested.

Merleau-Ponty^(15)^ conceives the body’s experience as a field of meaning-making, where sensations are intrinsically linked to movement. Each perceptual act enables the creation of meaning, opening space for new interpretations of different existential situations.

By practicing mindfulness, participants directed their attention fully to the sensations and perceptions present in their bodies and immediate environment^(11,31)^. This practice enabled a deeper connection with bodily experience, allowing greater awareness and understanding of the physical pains and tensions that arose during the postpartum period, and leading to beneficial changes.

Mindfulness fostered self-regulation, attentional control, emotion regulation, a change in perspective on the self, and increased body awareness. These psychological processes are considered essential in the postpartum period, since maternal distress can adversely affect the mother-infant relationship as well as interactions with the surrounding environment^(26,30)^.

Within a phenomenological framework, postpartum women’s reports of change after the mindfulness practice can be understood in light of Husserl’s concept of intentionality. Phenomenology seeks to understand subjective experience and the relation between consciousness and the world, emphasizing intentionality as a fundamental structure of consciousness^(17,31)^.

Such complementary therapeutic approaches have the potential to enhance women’s experience and overall well-being by changing how the phenomenon is perceived and lived and by enabling the construction of new meanings. Negative emotions-fear, pain, despair, and guilt-reported by participants P3, P10, and P19 were reframed, giving way to hope, calm, and closer affective bonds with their infants^(7,32)^.

By becoming aware of their physical and emotional sensations, postpartum women engaged in a reflective dialogue with themselves, exploring and making sense of their maternal experiences more deeply. From a phenomenological standpoint, Husserl^(31)^ and Merleau-Ponty^(15)^ argue that this link between bodily awareness and emotional self-regulation underscores the central role of awareness in experiencing and interpreting the phenomenonon.

Participants reported emotional relaxation and relief from psychological tension following the mindfulness sessions. Mindfulness-based stress-reduction programs may improve stress levels and coping skills, reduce perceived fear, improve sleep, and decrease anxiety and worry^(33,34)^.

Through mindfulness practice, participants directed their attention to the present moment and accepted the difficulties and challenges of the postpartum phase. According to Husserl^(35)^, such acceptance can enable a reappraisal of emotions and experiences. As the accounts indicate, postpartum women began to experience the present moment with greater awareness.

### Study limitations

The study was limited by being conducted during the COVID-19 pandemic, which imposed some restrictions on its implementation. Another limiting factor was that participants were discharged before completing all stages of the intervention, which excluded them from the study’s final results.

### Contributions to the field

This research provides support for rethinking and reformulating public policies and clinical care protocols regarding the care offered to women across the pregnancy-postpartum cycle. It shows that, through the practice of mindfulness, biopsychosocial adverse outcomes resulting from risk-related phenomena can be reduced. In addition, it offers health professionals a new evidence-based strategy for coping and care in emergency settings.

## FINAL CONSIDERATIONS

The study highlighted that the postpartum phase, in adverse situations, is marked by multiple changes that affect women as biopsychosocial beings, triggering psychological distress. Hospitalization and treatment, although intended to restore the health of the baby and/or the mother, also result in physical, emotional, and relational strain.

This study found that mindfulness techniques help minimize the emerging afflictions of the puerperal phase by reconnecting women with their bodies and emotions without judgment. This intervention created space for new possibilities, assisting them in reducing stress, adapting to the particularities of perinatality, and preventing additional complications.

The findings revealed that the practice of mindfulness directly contributed to reduced crying and anxiety, improved sleep quality, diminished guilt, and strengthened the bond between mother and baby. Moreover, participants reported greater body awareness, emotional self-regulation, and a sense of physical lightness. These experiences were expressed in interviews through accounts that demonstrated the reframing of the puerperal experience in the face of pain, guilt, and hospitalization.

These results underscore the relevance of mindfulness as a care strategy in hospital settings, especially for women facing emotional and clinical vulnerability. Including mindfulness in postpartum care protocols for women at risk is believed to promote physical, emotional, and interpersonal well-being, making the experience healthier and less debilitating.

Analyzing these experiences through the theoretical-methodological lens of phenomenological thought allows a deeper understanding of women’s experiences in their lived world. This study emphasizes the importance of ensuring a differentiated focus on postpartum women during the hospitalization of the mother-baby dyad for health reasons. Therefore, it suggests the development of further research and actions that may help mitigate the suffering experienced by these women.

## Data Availability

The research data are available only upon request.
