# Supplementary material for: Mindfulness practices for alleviating distress among postpartum women
Source: Rev Bras Enferm. 2025 Dec 8;78(Suppl 3):e20240525. doi: 10.1590/0034-7167-2024-0525 (PMC12707845; doi:10.1590/0034-7167-2024-0525)
Supplement: Translation

# Translation: Práticas de *mindfulness* na superação das inquietações da mulher no puerpério

**DOI:** 10.1590/0034-7167-2024-0525pt

**Authors:** Fernanda Veras Vieira Feitosa, Raimunda Magalhães da Silva, Waleska Benicio de Oliveira Carvalho, Débora Pereira Paixão, Marta Maria Soares Herculano, Maria Helena Carvalho Valente Presado, Vânia Krisna de Oliveira Albuquerque, Laura Pinto Torres de Melo

**Affiliations:** I Universidade de Fortaleza. Fortaleza, Ceará, Brasil; II Universidade Federal do Ceará. Fortaleza, Ceará, Brasil; III Escola Superior de Enfermagem de Lisboa. Lisboa, Portugal; IV Universidade Federal do Ceará, Empresa Brasileira de Serviços Hospitalares. Fortaleza, Ceará, Brasil

**Keywords:** Mindfulness, Puerpério, Mulher, Resiliência Psicológica, Saúde Mental.

## Abstract

**Objetivos:**

compreender as implicações da prática de *mindfulness* na superação das inquietações de puérperas internadas em uma maternidade pública.

**Métodos:**

trata-se de um estudo de intervenção realizado em uma maternidade entre março e junho de 2022, com 19 puérperas. Coletaram-se os dados por entrevistas aplicadas antes e depois de oito intervenções de *mindfulness*. Os resultados foram organizados e analisados mediante a análise temática.

**Resultados:**

a prática promoveu nas puérperas uma autorregulação psicológica, controle da atenção, regulação emocional, mudanças na perspectiva de si e maior consciência corporal. Além disso, minimizou temores, angústias e o estresse.**Considerações Finais:** ao despertar a consciência para as sensações físicas e emocionais de seus corpos, as puérperas envolveram-se em um processo reflexivo, que facilitou a superação das inquietações provenientes do puerpério.

## INTRODUÇÃO

Mundialmente, a cada minuto ocorre uma morte materna, totalizando cerca de 600 mil óbitos anuais. Em países de alta e média renda, a taxa de mortalidade é superior a 12/100 mil nascidos vivos, enquanto em países de baixa renda, chega a 239/100 mil. Para combater essa realidade, a Organização das Nações Unidas (ONU) e a Organização Mundial da Saúde (OMS) propuseram metas globais por meio dos Objetivos de Desenvolvimento Sustentável (ODS), a serem alcançados até 2030, visando reduzir a mortalidade materna, neonatal e os agravos no ciclo gravídico-puerperal^(1)^.

Para Castiglioni^(2)^, a fase puerperal é uma etapa do ciclo gravídico-puerperal que simboliza uma transição para a prática da maternidade e a regressão corpóreo-gravídica. Esse período é caracterizado por inúmeras transformações corporais e adaptações afetivas, podendo resultar em adversidades que envolvem o vínculo entre mãe e filho.

Nesse período, a mulher enfrenta conflitos entre as expectativas da gravidez e as mudanças após o parto, o que gera vulnerabilidades emocionais. O puerpério é marcado pela atenção a enfermidades não obstétricas, incluindo transtornos mentais e doenças crônicas não transmissíveis, que impactam significativamente a vida da puérpera^(3,4)^.

Dessa forma, quando ocorrem intercorrências, antes ou depois do nascimento, a maternidade pode ser interrompida ou alterada devido ao estresse físico e psicológico. O resultado são mudanças nos cuidados prestados aos filhos e na forma de a mulher ser e estar diante de si e do meio onde se encontra inserida, sendo necessários ajustamentos internos e externos para um melhor enfrentamento do momento vivenciado^(5)^.

Nesse contexto, têm ocorrido progressos na busca e no desenvolvimento de alternativas terapêuticas voltadas para mitigar os efeitos do puerpério e suas complicações. Entre essas alternativas, destacam-se as Práticas Integrativas e Complementares (PICs), que proporcionam um cuidado integral à população, englobando fitoterapia, acupuntura, homeopatia, *mindfulness*, termalismo, entre outras. Instituídas em 2006 por meio do Plano Nacional de Práticas Integrativas e Complementares, passaram a somar 29 modalidades em 2018^(6)^.

O *mindfulness* pode ser oportuno e eficaz, pois é uma técnica de observação consciente que minimiza a geração de sentimentos ou pensamentos perniciosos, os quais repercutem na saúde. A prática busca promover o autocontrole por meio da atenção plena, uma forma de meditação que auxilia no equilíbrio emocional e na autopercepção do corpo. Ela oportuniza ganhos na qualidade do sono e no bem-estar psicológico, físico, inter-relacional e outros^(7-9)^.

Em países desenvolvidos, essa prática tem sido aplicada em diversos contextos clínicos, proporcionando benefícios tanto para o corpo quanto para a mente. É uma técnica amplamente utilizada para promover mudanças comportamentais e tem demonstrado efeitos positivos no comportamento de adultos com diferentes condições de saúde^(10,11)^.

Diante das diversas nuances inerentes ao puerpério e das implicações na saúde da mulher após as intercorrências presentes nessa fase, acredita-se que o *mindfulness* pode ser um instrumento significativo nesse processo. Com isso, questionou-se sobre as mudanças que sua prática promove em puérperas internadas em uma maternidade.

Apesar do crescente reconhecimento dos benefícios do *mindfulness* no campo da saúde mental, observa-se uma escassez de estudos qualitativos que investiguem essa prática durante o puerpério em contextos hospitalares de alto risco no Brasil. Pouco se conhece sobre as experiências vividas por puérperas hospitalizadas após intervenções baseadas em *mindfulness*, especialmente considerando os aspectos emocionais, corporais e relacionais nesse período. Essa lacuna compromete a construção de práticas de cuidado centradas na mulher e integradas às demandas específicas da perinatalidade em situações adversas.

Com os resultados alcançados, espera-se que surjam novas reflexões e considerações sobre o cuidado e atenção à saúde da puérpera. Esta pesquisa gera subsídios para repensar novas políticas e reexaminar aquelas voltadas ao cuidado integral e bem-estar da mulher, favorecendo um ambiente de segurança, respeito e assistência integral.

## OBJETIVOS

Compreender as implicações da prática de *mindfulness* na superação das inquietações de puérperas internadas em uma maternidade pública.

## MÉTODOS

### Aspectos éticos

O estudo foi aprovado pelo Comitê de Ética em Pesquisa da instituição onde a pesquisa foi realizada. Foram asseguradas as recomendações éticas referentes à pesquisa que envolve seres humanos, conforme previsto nas Resoluções 466/12 e 510/2016 das Ciências Sociais e Humanas, do Conselho Nacional de Saúde (CNS)^(12,13)^.

Após a anuência da instituição, as participantes foram contatadas presencialmente, receberam esclarecimento sobre os princípios da pesquisa e assinaram o Termo de Consentimento Livre e Esclarecido. Devido ao fluxo constante de novas admissões, os esclarecimentos foram oferecidos continuamente. Vale ressaltar que nenhuma puérpera convidada recusou participar do estudo nem tinha vínculo com as pesquisadoras.

A fim de assegurar o sigilo de cada relato, foi usada a letra inicial do grupo estudado (“P” de “puérpera”), seguida de número indicando a ordem de realização da entrevista. Os princípios empregados para finalizar cada interlocução foram o de saturação, deleite e apreensão das falas expressas pelas puérperas.

### Referencial teórico-metodológico

A pesquisa fundamentou-se nos preceitos teóricos e metodológicos da fenomenologia, com foco nos conceitos de Edmund Husserl^(14)^ e Merleau-Ponty^(15)^.

Conforme Husserl, a “experiência” é uma forma de interação que o sujeito tem com os objetos no seu ambiente. Essa forma de relação não deve ser considerada apenas como manifestações de estados mentais internos, pois é sempre intencional e direcionada a algo externo, ao qual referiu-se como “fenômeno”, ou seja, manifestações da consciência perante o contexto em que está imersa^(16)^.

Merleau-Ponty^(15)^ direciona o olhar para a experiência por intermédio da percepção. Para esse autor, a percepção restaura a lógica de vida e abre espaço para acessar o mundo no qual se participa. São nos processos da compreensão que surgem os significados primordiais da verdade latente da existência do vivido. Perceber é, pois, uma ação, um conhecer pragmático perante o fenômeno.

Dessa forma, Merleau-Ponty^(15)^ postula que as pessoas estão imersas no mundo por meio do corpo, visto que é através dele que elas se percebem e vivenciam. O corpo é a base dos sentidos, ou seja, a construção dos sentidos das relações existentes do ser com o mundo. Todavia, o ser se percebe em sua totalidade ao estruturar-se por meio da relação com seu entorno, o que, por conseguinte, reflete-se no próprio entorno. Ao compreender as pessoas como corpo compenetrado no mundo, ressalta-se a importância de vivenciar a experiência perceptiva, acreditando que o saber começa no corpo e na relação com o mundo vivido.

### Tipo de estudo

Trata-se de um estudo de intervenção, com abordagem qualitativa. Seguiu os princípios do *Consolidated Criteria for Reporting Qualitative Research* (COREQ), delineados para a apresentação de pesquisa qualitativa. Nesse método, são coletados dados não quantificáveis com base nas experiências vividas pelos participantes e captados por meio das características subjetivas de cada pessoa envolvida no cenário estudado^(17,18)^.

### Procedimentos metodológicos

Para a aplicação da técnica *mindfulness*, o agrupamento das puérperas se deu com quatro a dez participantes, número postulado por Zhang^(5)^ como favorável para conduzir processos grupais. Além disso, favorece a abertura para o espaço de fala, proporcionando um melhor entendimento e apreensão dos fenômenos expressos. Cada puérpera participou de oito sessões de *mindfulness*.

É importante pontuar-se que, dentre as intervenções baseadas em *mindfulness*, as mais usadas são a Terapia Cognitiva Baseada em *Mindfulness* (MBCT) e a Terapia Comportamental Baseada na Aceitação (ABBT). Este estudo fundamentou-se no Programa de Redução de Estresse Baseado em *Mindfulness* (MBSR), estruturado em oito sessões e projetado por Kabat-Zinn^(19-21)^.

As práticas de *mindfulness* foram oferecidas três vezes por semana nos turnos da manhã e da tarde, seguindo uma programação pré-elaborada para os encontros. A seguir, são descritos cada um dos encontros.

No primeiro momento, trabalhou-se com a Prática de Consciência Plena na Respiração (realização da prática com foco na respiração): essa técnica utiliza como âncora a própria respiração. Trata-se de orientar a atenção às experiências e sensações de “respirar sabendo que se está respirando”, ou seja, com consciência plena, momento a momento^(22)^.

Na segunda intervenção, aplicou-se a Prática de Escaneamento Corporal (elaboração do escaneamento do corpo), buscando ancorar a atenção em qualquer sensação que possa se manifestar no corpo em repouso. Nessa técnica, podem surgir sensações agradáveis, desagradáveis ou neutras: pode-se acolhê-las, permitindo que surjam e desapareçam, cultivando atitudes de abertura, curiosidade e gentileza, fundamentais em *mindfulness* ^(19)^.

No terceiro encontro, usou-se a Prática de Caminhada Meditativa (despertar do aqui e agora durante a caminhada): procura-se cultivar a atenção focada no corpo em movimento e perceber como é caminhar sem ter um objetivo de chegar a algum lugar^(20)^.

Na quarta intervenção, adotou-se a Prática de Monitoramento Aberto (reflexão sobre as relações familiares e sociais): consiste em testemunhar o surgimento, manifestação e cessação de eventos mentais em nosso campo de experiência, desenvolvendo a habilidade de observar eventos mentais sem se apegar ou ter aversão a eles^(23)^.

No quinto encontro, executou-se a Prática de Movimentos com Plena Consciência (relação do ambiente familiar e hospitalar): tem a intenção de observar as respostas emocionais ao corpo, as necessidades (impulsos) de escapar do desconforto, o autojulgamento e a avaliação^(21)^.

No sexto momento, trabalhou-se com a Prática de Interdependência (reflexão sobre a relação entre passado, presente e futuro): busca-se ampliar a motivação para expandir o círculo de cuidado, estabelecendo conexões e diminuindo a postura de indiferença a outros seres, bem como cultivando gratidão e contentamento diante da existência^(22)^.

No sétimo encontro, aplicou-se a Prática da Amorosidade (relação consigo mesmo e com o outro): tem a finalidade de favorecer um espaço que conecte as pessoas com seus corações e traga à consciência o fato de nos importarmos uns com os outros^(20)^.

A oitava intervenção se deu por meio da Prática de Reconhecimento da Humanidade Compartilhada, com votos de Boa Vontade (reflexões sobre as relações consigo, com o outro e com o mundo): busca aumentar a sensação de empatia, conexão e pertencimento e cultivar as atitudes de boa vontade^(20,22)^.

### Cenário do estudo

Realizou-se este estudo em uma Casa da Gestante, Bebê e Puérpera (CGBP) de uma maternidade pública de nível terciário, especializada em obstetrícia de alto risco. A CGBP tem como objetivo servir como uma residência de cuidados e acompanhamento especializados para gestantes, puérperas e recém-nascidos de alto risco que ainda precisam de cuidados em saúde diariamente, mas sem necessidade de internação hospitalar.

O local funciona como um anexo da maternidade, dispondo de uma ampla equipe multiprofissional para prestação de cuidados especializados. A equipe conta com médicos, enfermeiros, técnicos de enfermagem, psicólogos, fisioterapeutas, entre outros profissionais que atuam em sobreaviso.

### Fonte de dados

Participaram da pesquisa 19 puérperas selecionadas com base nos critérios de amostragem por conveniência. Foram incluídas mulheres na fase puerperal, a partir de 18 anos, que puderam permanecer na unidade por um período de dez dias^(10)^, a fim de participarem da intervenção de oito sessões de *mindfulness* e das entrevistas inicial e final. Excluíram-se as participantes que necessitavam de repouso absoluto por ordens médicas e as que não puderam participar de todas as etapas da pesquisa.

Para as entrevistas, reservou-se um tempo para um diálogo informal com as participantes, buscando romper as possíveis barreiras presentes. Todavia, essas conversas ocorreram brevemente devido à existência de uma programação nos serviços onde a pesquisa foi realizada.

### Coleta e organização dos dados

Os dados foram coletados entre março e junho de 2022 por meio de uma entrevista semiestruturada para obter informações sociodemográficas, clínicas e obstétricas, bem como sobre estilo de vida. A pesquisadora psicóloga e duas colaboradoras treinadas em pesquisa de campo efetuaram a coleta.

A fase de levantamento dos dados deu-se em três etapas: a primeira, por intermédio de uma entrevista inicial, que incluiu perguntas guiadas sobre as vivências no puerpério e expectativas com a prática de *mindfulness*; a segunda envolveu a participação em oito^(8)^ sessões dessa técnica, em estágios progressivos e definidos com duração aproximada de 60 minutos cada intervenção, sendo mediada de forma grupal em ambiente preparado com até 10 mulheres; e a terceira etapa consistiu em uma entrevista com questões sobre as experiências após a técnica e suas implicações. As duas entrevistas (antes e depois da intervenção) aconteceram com as 19 participantes, ou seja, sem desistência.

As entrevistas foram gravadas em áudio, de forma individual, sendo a entrevista inicial realizada antes da intervenção e a entrevista final realizada após a intervenção, com duração de 20 a 60 minutos, respeitando a necessidade de cada entrevistada. Assegurou-se a confiabilidade das respostas, que foram posteriormente transcritas na íntegra e reapresentadas às entrevistadas.

### Análise dos dados

Analisaram-se os achados pela análise temática, para estruturar os informes coletados por meio de pré-análise, exploração e interpretação dos discursos. Buscaram-se colocações convergentes e diferenciadas, contudo representativas de significado para as puérperas. Com a exploração do material coletado, realizaram-se leituras precisas para encontrar as unidades de significado diante do fenômeno vivenciado^(18)^. A interpretação dos resultados fundamentou-se nas abordagens fenomenológicas.

O conteúdo analisado organizou-se em três temáticas: “Expectativas de mudanças com a prática de *mindfulness*”, “Implicações no corpo e na mente com a prática de *mindfulness*” e “*Mindfulness* na superação das inquietações da puérpera diante do fenômeno vivido”.

## RESULTADOS

Das 19 puérperas participantes da pesquisa, constatou-se que 35% residiam no município de Fortaleza; e 65%, na região metropolitana. Situam-se na faixa etária de 18 a mais de 36 anos, com predomínio da faixa etária de 21 a 25 anos (31,57%). Acerca dos diagnósticos, 84,21% foram reagentes ao teste *Venereal Disease Research Laboratory* (VDRL), e 68,42% dos recém-nascidos apresentaram sífilis congênita. Em relação aos dados obstétricos, 52,6% eram multíparas; e 47,3%, primíparas. Quanto ao tipo de parto, 78,9% foram cesáreas, enquanto 21,06% foram partos vaginais.

No tocante aos sentimentos sobre a internação, 36,8% das puérperas sentiram-se satisfeitas; 36,8%, com medo; e 15,7%, apreensivas. Quando perguntadas sobre a prática de atividades para reduzir o estresse e promover o bem-estar, 25% afirmaram fazê-lo, enquanto 75% não praticavam.

### Expectativas de mudanças com a prática de *mindfulness*

Essa temática abordou as expectativas que as puérperas tiveram sobre a prática de *mindfulness*, como o desejo de lidar melhor com as transformações psíquicas e corporais vividas no puerpério em um cenário de risco.

*Foi planejado, mas um baque, o bebê doente* [...]. *Espero que essa prática ajude a acalmar meu corpo e me distraia. Fico pensando no meu filho direto, isso me deixa cansada. Minha mente necessita de calma.* (P1)

*Planejei a gravidez, não estava preparada para um parto prematuro. O puerpério está sendo muito difícil. Minha mente está fora do lugar, meu corpo não responde aos comandos da minha cabeça. Anseio que essa técnica funcione.* (P6)

*Estou ansiosa para que tudo isso passe. Espero que essa técnica me ajude a acalmar e encontrar paz.* (P10)

[...] *Preciso que o corpo seja cuidado. Tenho vivido dificuldades e acredito que isso vai ajudar a superar muitas delas. Anseio ter uma mente calma e um corpo sem tensões.* (P17)

Elas almejavam que a prática de *mindfulness* abrisse um espaço para mudanças que vão além do corpo e da mente, ajudando nas relações familiares, consigo mesmas e na internação, conforme percebido nas seguintes falas:

*Acredito que relaxe a mente, que melhore meu relacionamento com meu esposo e comigo mesma, pois preciso superar coisas dentro de mim.* (P8)

*O medo envolve minha mente e corpo, pela culpa da contaminação do bebê. Desejo que* mindfulness *me ajude a superar isso, que olhe para ele sem rejeição.* (P5)

*Espero que traga benefícios, relaxe o corpo, alivie as dores e que nos deixe mais calmas, porque às vezes bate desespero, me sinto culpada e revoltada com todos.* (P12)

*Preciso me organizar, pois não me sinto a mesma mulher: a relação com a minha família mudou. Acho que a internação e essa doença mexeu comigo; preciso que essa atividade funcione.* (P16)

### Implicações no corpo e na mente com a prática de *mindfulness*

Essa temática abordou os relatos das puérperas após vivenciarem a prática de *mindfulness* em sua existência. Foi percebido que a técnica pode proporcionar diversos benefícios, permitindo que o sofrimento e as tensões emergidas no ciclo puerperal encontrem um espaço de tranquilidade.

Por exemplo, o choro intenso pode ser acalmado não por falta de motivo, mas pela descoberta de novas possibilidades de vida, reduzindo a ansiedade e o peso da situação. Isso permite desconectar-se de pensamentos invasivos e focar a atenção em um estado de calma, como observado nos seguintes relatos:

*São inúmeros os benefícios, como a tranquilidade da mente, alívio das tensões e estresse enfrentados nesses dias difíceis. O corpo parece leve, o choro se acalma e silencia, parei de pensar no pior.* (P5)

*Essa prática realmente traz benefícios para a mente e corpo. As tensões diminuíram, os inchaços melhoraram significativamente. As técnicas de respiração tiraram um peso de mim. É incrível como faz uma grande diferença em meu corpo. O choro, que antes era incontrolável, parece ter desaparecido.* (P3)

*Essa prática me acalma muito, desviando o foco de coisas negativas e retirando um peso de mim. Sinto que ela alivia o peso da minha consciência: relaxo.* (P11)

Devido à situação vivida, as puérperas revelaram que sentiam aversão ao bebê, causando sofrimento e sentimento de culpa. No entanto, relataram que, após as intervenções, houve melhora na relação com o filho, aproximando-as e modificando a forma como se relacionavam com ele.

[...] *não dormia durante a noite; após essas palestras, comecei a dormir. Agora estou calma, muito relaxada. Minha relação com meu bebê melhorou. Ele fica tranquilo quando praticamos. Antes, havia algo estranho: não o aceitava, me culpava por isso.* (P13)

[...] *no início, eu sentia repulsa pelo meu bebê, o que me causava dor. Mas após essas atividades, percebi que as coisas mudaram.* (P12)

[...] *agora consigo dormir muito melhor, consigo me relacionar com minha filha sem culpa, isso é maravilhoso. Essa prática realmente mudou muitas coisas em mim.* (P15)

Destaca-se que o *mindfulness* ajuda a diminuir as dores físicas. As puérperas mencionaram inchaço e dores corporais após o parto, resultantes de tensões e mudanças típicas dessa fase. A prática de *mindfulness* mostra-se integralmente benéfica: tanto na redução desse desconforto quanto na sensação de calma diante da situação.

*Essas atividades foram um divisor de águas, abriram novos caminhos para viver a vida e lidar com as situações de forma mais suave, reduzindo os medos.* (P6)

[...] *o corpo relaxa: as tensões e dores diminuíram. Os inchaços melhoraram. Acredito que tenha ajudado na circulação. Agora, pensando na minha mente, como melhorou! Estava muito aflita.* (P8)

[...] *tantas coisas mudaram em mim, além do que podia imaginar. Meu corpo e meus pensamentos foram afetados por esses processos.* (P12)

### *Mindfulness* na superação das inquietações da puérpera diante do fenômeno vivido

Essa temática abordou questões relacionadas ao uso do *mindfulness* para o enfrentamento dos temores e da insegurança vivenciados no ciclo puerperal. As puérperas relataram uma diminuição do medo, da angústia e do estresse, bem como revelaram a atribuição de novos significados ao fenômeno vivenciado.

*Minha mente se acalmou, e meu corpo, tão estressado, encontrou um lugar de tranquilidade em meio a tantos impasses e angústias.* (P8)

*Estava muito agitada, sem rumo, com medos. Aos poucos, as minhas emoções foram encontrando um lugar de paz dentro de mim* […] *me acalmei.* (P12)

*Quando meditamos, nos sentimos melhores e mais felizes mesmo imersas em tanta dor.* (P13)

O medo, a dor e a desesperança deram lugar à esperança e a uma conexão mais profunda com o filho, conforme as falas:

*Percebi que essas emoções só me atrapalhavam. O* mindfulness *está sendo muito bom para enfrentar os medos que estamos passando.* (P10)

*Nesses dias, muitas coisas ganharam um novo sentido para nós. Passei a olhar para meu bebê sem medo e dor, mas com olhos de esperança, acreditando que ficaria bem.* (P3)

*Depois desses dias, estou mais perto do meu bebê. Olho para ele sem culpa, aproveito mais. Aprendi a me acalmar e deixar o medo e a culpa de lado, viver com novos olhos.* (P19)

## DISCUSSÃO

No pós-parto, as mulheres enfrentam sofrimento psicológico devido a mudanças físicas, aos cuidados com o bebê e aos ajustes pessoais e profissionais. Em gestações de risco, o desconforto psíquico é intensificado, levando a um aumento de ansiedade, estresse e medo^(24,25)^. Essa situação é uma razão significativa para o desenvolvimento do luto materno, conforme observado nas colocações das participantes.

Schiele^(25)^ e Araújo^(10)^ argumentam que as preocupações relacionadas a problemas de saúde, perda de controle, isolamento, distanciamento do ambiente familiar, desconforto físico e procedimentos obstétricos, muitos dos quais invasivos física e mentalmente, são fatores geradores de sentimentos adversos.

Essas emoções seguem os princípios de Merleau-Ponty^(15)^: a experiência é inerente ao indivíduo e não pode ser totalmente reprimida, mesmo que ele tente. Segundo o autor, há mais na experiência do que pode ser expresso em pensamentos, gestos e palavras. A natureza da experiência é ambígua, envolvendo emoções relacionadas a aspectos que não podem ser completamente definidos, exigindo uma interação aberta entre corpo, mente e alma.

Nessa conjuntura, conforme afirmado por da Silva et al.^(26)^ e Porter et al.^(9)^, o *mindfulness* pode contribuir no enfrentamento dos desafios do puerpério, já que direciona as pessoas a prestarem atenção plena ao presente, sem julgamentos, percebendo ou conscientizando-se do corpo no momento vivido. Klainin-Yobas et al.^(27)^ e Santos et al.^(28)^ afirmam que a resiliência psíquica é um processo dinâmico: envolve mudanças e permite às pessoas encontrarem equilíbrio e prosperarem em suas relações consigo mesmas, com os outros e com o mundo. A resiliência é um produto da interação humana com o ambiente.

Os participantes desta pesquisa compartilham experiências em que houve o aprimoramento da resiliência mental e física diante de adversidades. Segundo Merleau-Ponty^(15)^, a intencionalidade do corpo está relacionada ao mundo concebido, refletindo desejos e sentidos formados ao longo da vida e imersos em perspectivas simbólicas vivenciadas pelo ser humano.

O uso de intervenções baseadas em *mindfulness* (MBIs) em ambientes clínicos têm crescido, com pesquisas destacando seus efeitos positivos na saúde. Conforme da Silva et al.^(26)^, tais intervenções são eficazes na redução da ansiedade, estresse e depressão durante o período perinatal. As experiências das entrevistadas corroboram essa observação, visto que relataram redução significativa no estresse percebido e aumento na atenção plena.

As puérperas mencionaram sentir calma mental, menos angústia e menos choro, destacando a importância do *mindfulness* para promover bem-estar e saúde mental no período perinatal. Segundo Wang at al.^(29)^, essa prática ajuda as pessoas a serem conscientes de seus pensamentos e sentimentos, permitindo uma relação mais positiva com eles. Isso facilita a autoaceitação e a capacidade de lidar com condições de saúde desafiadoras, como internações ou doenças graves.

O corpo constrói seus estados de conhecimento e seus sentidos corporais de acordo com a maneira pela qual vivencia o presente em sua experiência. Assim, independentemente do modo como se expressa, o corpo incorpora seus sentidos e dá forma à singularidade de cada ser em sua existência^(15,30)^.

Merleau-Ponty^(15)^ concebe as mudanças comportamentais no corpo como um simbolismo do próprio existir. Segundo sua perspectiva, o corpo renuncia a si mesmo, torna-se opaco e experimenta sensações e emoções que normalmente seriam reprimidas. Quando uma mãe imagina as situações enfrentadas por seu filho durante o tratamento da doença, ela vivencia sensações distintas em seu corpo. Nesse momento, o corpo é o local onde sua existência se manifesta.

Desse modo, Merleau-Ponty^(15)^ conceitua a experiência do corpo como um campo criador de sentidos, onde as sensações estão intrinsecamente ligadas ao movimento. Cada ato perceptivo possibilita uma criação, abrindo espaço para novas interpretações das diferentes situações existenciais.

Ao praticar *mindfulness*, as participantes direcionaram sua atenção plenamente para as sensações e percepções presentes em seus corpos e no entorno imediato^(11,31)^. Essa atividade viabilizou uma conexão mais profunda com as experiências corporais, possibilitando uma conscientização e compreensão das dores e tensões físicas que surgiram durante o puerpério, o que resultou em mudanças favoráveis.

O *mindfulness* promove a autorregulação psicológica, controle da atenção, regulação emocional, mudanças na perspectiva de si e maior consciência corporal. Esses processos psicológicos são vistos como essenciais no puerpério, pois o sofrimento materno pode afetar adversamente a relação entre a mãe e a criança, bem como com o meio^(26,30)^.

No contexto da fenomenologia, os relatos das puérperas sobre as mudanças percebidas com a prática de *mindfulness* podem ser compreendidos à luz do conceito de “intencionalidade” de Husserl. A fenomenologia busca compreender a experiência subjetiva e a relação entre a consciência e o mundo circundante, enfatizando a importância da intencionalidade como estrutura fundamental da consciência^(17,31)^.

Essa abordagem terapêutica complementar tem o potencial de contribuir para uma experiência mais positiva e para o bem-estar geral, proporcionando mudanças na maneira de enxergar e vivenciar o fenômeno, buscando novos significados^(7,32)^. Os sentimentos negativos como medo, dor, desesperança e culpa apontados pelas participantes P3, P10 e P19 foram ressignificados, abrindo espaço para novos sentidos como esperança, calma e estreitamento de laços afetivos com seus filhos.

Ao tornarem-se conscientes das sensações físicas e emocionais, as puérperas puderam envolver-se em um diálogo reflexivo consigo mesmas, explorando e compreendendo suas experiências maternas de forma mais profunda. Segundo Husserl^(31)^ e Merleau-Ponty^(15)^, essa conexão entre a consciência corporal e a autorregulação emocional, dentro da perspectiva fenomenológica, enfatiza a importância da consciência como elemento central no processo de vivência e compreensão das experiências do fenômeno.

As puérperas entrevistadas relataram sentir um relaxamento emocional e um alívio das tensões psicológicas após as sessões de *mindfulness*. Programas de redução de estresse baseados nessa técnica podem melhorar os níveis de estresse e as habilidades de enfrentamento, reduzir a percepção de medo, melhorar o sono e diminuir a ansiedade e preocupação^(33,34)^.

Por meio da prática de *mindfulness*, as puérperas puderam direcionar sua atenção plenamente ao momento presente, aceitando as experiências vividas, dificuldades e desafios da fase puerperal. Segundo Husserl^(35)^, essa aceitação permitiu uma ressignificação das emoções e experiências. Conforme expresso nos relatos, as puérperas passaram a vivenciar o momento de forma consciente.

### Limitações do estudo

O estudo limitou-se por ter sido realizado durante a pandemia de covid-19, gerando algumas restrições em sua execução. Outro aspecto limitante foi o fato de que as participantes receberam alta antes de completar todas as fases da intervenção, o que as excluiu dos resultados finais deste estudo.

### Contribuições para a Área

Esta pesquisa fornece subsídios para repensar e reformular políticas públicas e protocolos assistenciais acerca do cuidado prestado às mulheres no ciclo gravídico-puerperal. Mostra que, por intermédio da prática de *mindfulness*, podem-se reduzir as intercorrências biopsicossociais acarretadas pelos fenômenos de risco vivenciados. Ademais, proporciona aos profissionais de saúde uma nova estratégia de enfrentamento e cuidado baseada em evidências, considerando cenários emergenciais.

## CONSIDERAÇÕES FINAIS

O estudo destacou que a fase puerperal em situações adversas é permeada por múltiplas mudanças que afetam as puérperas enquanto seres biopsicossociais, desencadeando sofrimentos psíquicos. O fenômeno da hospitalização e do tratamento, embora seja em prol da recuperação da saúde do bebê e/ou puérpera, leva a desgastes físicos, emocionais e relacionais.

Neste estudo, constata-se que a técnica de *mindfulness* minimiza as aflições emergentes na fase puerperal, conectando as puérperas com seus corpos e emoções, sem julgamentos. Essa intervenção abriu espaços para novas possibilidades, ajudando-as na redução do estresse e na adaptação às diversas particularidades da perinatalidade e diminuindo o surgimento de novos agravos.

Os achados revelaram que a prática de *mindfulness* contribuiu diretamente para a diminuição do choro e da ansiedade, melhora na qualidade do sono, redução da culpa e fortalecimento do vínculo entre mãe e bebê. Além disso, as participantes relataram maior consciência corporal, autorregulação emocional e sensação de leveza física. Essas vivências foram expressas nas entrevistas por meio de falas que evidenciaram a ressignificação da experiência puerperal diante da dor, da culpa e da internação.

Esses resultados justificam a relevância da prática como estratégia de cuidado no contexto hospitalar, especialmente para mulheres em situação de vulnerabilidade emocional e clínica. Acredita-se que a inclusão de *mindfulness* em protocolos de cuidado à puérpera em situações de risco possa promover o bem-estar físico, emocional e interpessoal, proporcionando uma experiência mais saudável e menos debilitante.

A análise dessas vivências sob a ótica teórico-metodológica do pensamento fenomenológico possibilita uma compreensão mais profunda das experiências das puérperas em sua interação com o mundo vivido. Este estudo evidencia a importância de assegurar um enfoque diferenciado às puérperas durante o período de hospitalização do binômio bebê-puérpera, por questões de saúde. Diante disso, sugere-se a elaboração de mais pesquisas e ações que contribuam para a minimização do sofrimento vivenciado por essas mulheres.

## Data Availability

Os dados de pesquisa só estão disponíveis mediante solicitação.
